# New insights into proteinuria/albuminuria

**DOI:** 10.3389/fphys.2022.991756

**Published:** 2022-09-26

**Authors:** Wayne D. Comper, Julijana Vuchkova, Kevin J. McCarthy

**Affiliations:** ^1^ Salaqua Diagnostics Inc, New York, NY, United States; ^2^ Department of Cellular Biology and Anatomy, LSU Health Sciences Center, Shreveport, LA, United States

**Keywords:** nephrotic syndrome, hypoproteinemia, retrieval of filtered proteins, Park and Maack proximal tubular cell receptors, charge selectivity, endothelium

## Abstract

The fractional clearance of proteins as measured in healthy human subjects increases 10,000–100,000- fold when studied in nephrotic patients. This remarkable increase cannot be accounted for by extracellular biophysical mechanisms centered at the glomerular filtration barrier. Rather, it is the nephron and its combination of filtration and cellular uptake that can provide a plausible explanation of these fractional clearance changes. The nephron has two regions that critically determine the level proteinuria/albuminuria. Glomerular filtration of plasma proteins is primarily a size selective event that is basically unchanged in acquired and genetic kidney disease. The glomerular concepts of ‘charge selectivity’ and of ‘large pores’, previously used to explain proteinuria, are now recognized to be flawed and non-existent. Filtered proteins then encounter downstream two protein receptors of the Park and Maack type associated with the proximal tubular cell. The high capacity receptor is thought to retrieve the majority of filtered proteins and return them to the blood supply. Inhibition/saturation of this pathway in kidney disease may create the nephrotic condition and hypoproteinemia/hypoalbuminemia. Inhibitors of this pathway (possibly podocyte derived) are still to be identified. A relatively small proportion of the filtered protein is directed towards a high affinity, low capacity receptor that guides the protein to undergo lysosomal degradation. Proteinuria in normoproteinemic states is derived by inhibition of this pathway, such as in diabetes. The combination of glomerular sieving, and the degradation and retrieval pathways can quantitatively account for the changes in fractional clearance of proteins in the nephrotic condition. Finally, the general retrieval of filtered protein by the proximal tubular cell focuses on the teleological importance of this cell as this retrieval represents the third pillar of retrieval that this cell participates in (it also retrieves water and salt).

## Introduction

Richard Bright in 1827 was one of the first to recognize that the appearance of protein in the urine was specifically due to kidney disease. What is remarkable that almost 200 years later a debate still continues as to whether proteinuria is a glomerular centric problem or a nephron problem.

The controversy is reflected even in recent publications. Benzing and Salant (Benzing and Salant, 2021) in the *New England Journal of Medicine* adopt a glomerular centric approach based on biophysical models of Haraldsson and Deen and others (Haraldsson et al., 2008). These models, however, have been extensively investigated and subsequently invalidated (Comper and Russo, 2009; Comper, 2022). Other considerations have incorporated both glomerular filtration and tubular uptake (Comper and Russo, 2009; Comper, 2014; Molitoris et al., 2022). This article reviews all these approaches contributing to proteinuria/albuminuria.

## Pathways for filtered albumin/protein processing through experimental studies *in vivo*

### Degradation pathway

**FIGURE 1:**
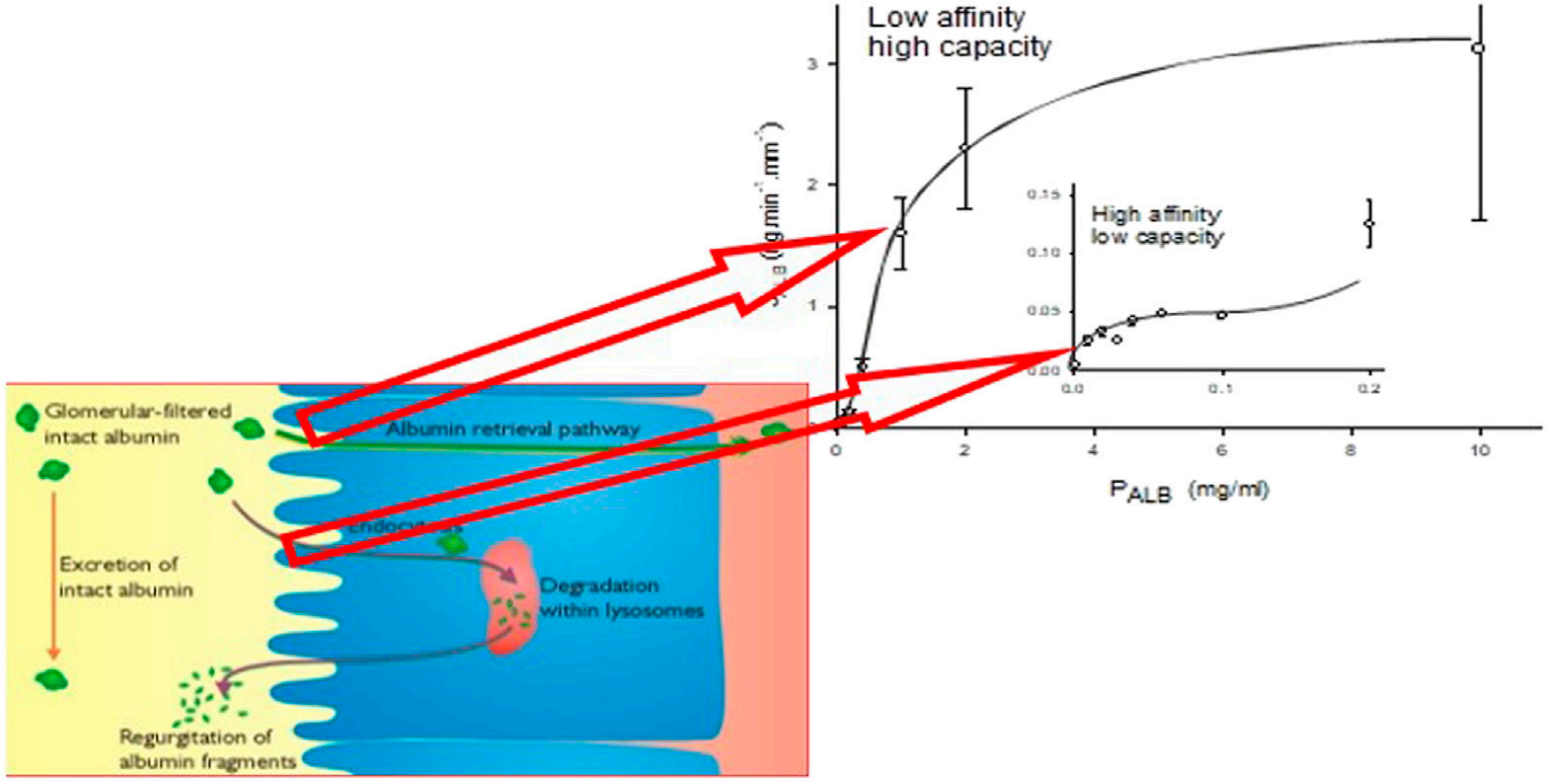
The line graph represents the kinetics of albumin absorption in rabbit proximal convoluted tubules from Park and Maack (Park and Maack, 1984). Endocytic uptake of albumin (J_ALB_) (ordinate) is plotted against perfusate concentrations of albumin (P_ALB_). Each of 57 tubules was perfused with a fixed P_ALB_ ranging from 0.0012 to 10 mg/ml. Values are means ± SE of at least 3 tubules. The absorption curve was analyzed in terms of Michaelis-Menten kinetics and has at least two components: an overall high-capacity/low-affinity system (J_ALB_^max^ = 3.7 ng min^−1^ mm tubule length^−1^, Michaelis-Menten constant K_m_ = 1.2 mg ml^−1^) and a low-capacity system (inset), which saturates near what was thought to be the physiological ranges of tubular fluid albumin concentrations as determined by micropuncture in mammals (J_ALB_^max^ = 0.0064 ng min^−1^ mm tubule length^−1^, K_m_ = 0.031 mg ml^−1^). The schematic represents depiction of the albumin retrieval and degradation pathways in the proximal tubular cell and the relationship of these pathways to the binding analysis of Park and Maack. More recent studies have suggested that these pathways occur for all filtered proteins with molecular weights >30 kDa (Comper et al., 2016).

One of the most striking results that is seen with the introduction of labelled albumin into the circulation is that the labelled material in the urine is mainly associated with small peptides (Figure 1) (Osicka et al., 1997; Osicka et al., 2000; Russo et al., 2002; Comper et al., 2008; Vuchkova and Comper, 2015). This is the result of filtered albumin being degraded by the proximal tubular cell (PTC). The extent of this degradation can be modulated by the disease process. For example, in diabetic patients the excreted labelled albumin can be predominantly intact and is accompanied by a smaller quantity of labelled peptides (Osicka et al., 2000) for the same amount of labelled material being excreted as in healthy controls. This inhibition of degradation is a characteristic of the disease process. The degradation pathway is quite non-specific; all labelled proteins studied undergo degradation.

### Retrieval pathway

The first and only demonstration so far of circulating plasma albumin being filtered and then returned to the renal vein (retrieval) was published in 1999 (Eppel et al., 1999). Retrieval was measured through the introduction of a small pulse of radioactive [^3^H]albumin into the rat renal artery *in vivo*, followed by examination of the radioactive profile from the renal vein effluent. It is viewed that the filtered portion of [^3^H]albumin in the pulse will be taken up by the PTC retrieval pathway, which will return the albumin to the blood supply undegraded (Figure 1). This recycled albumin appeared as a second peak(s) in renal vein effluent after the initial bolus had passed. Quantitation of the glomerular flux of albumin from the size of the second peak relative to the control (controls included either [^3^H]horseradish peroxidase, inulin labeled with radioactive carbon-14, or [^3^H]albumin in a nonfiltering kidney) accurately estimated the predicted glomerular flux of albumin governed by size selectivity alone; the calculated GSC was 0.04. This meant that this pathway was relatively high capacity as compared to the degradation pathway. Similar results were obtained using *ex situ* kidney perfusion.

The existence of the PTC retrieval pathway was confirmed 14 years later using a transgenic mouse with podocyte-specific expression of doxycycline-inducible tagged murine albumin (Tenten et al., 2013).

## Biphasic nature of albuminuria/proteinuria

The nephron then has two opportunities to govern the urinary excretion of proteins; one at the glomerular filtration level and the other further downstream in terms of PTC uptake. Based on the discussion below it is likely that glomerular filtration may be basically invariant for acquired and genetic kidney disease. With this in mind, we would suggest that one aspect that limits urinary protein excretion is PTC processing. Data in the literature is indicative that the process is biphasic which ensures that proteinuria/albuminuria is not a continuum. There are two major lines of evidence that support this; albumin uptake by perfused tubules and protein fractional clearances in diseased states.

Albumin binding and uptake to isolated rabbit perfused tubules with varying concentrations of albumin (Park and Maack, 1984) have identified two binding sites; a high affinity, low capacity site (Michaelis constant = 0.031 mg ml^−1^) and a low affinity, high capacity site (Michaelis constant = 1.2 mg ml^−1^) (Figure 1). These distinct sites have been associated with entirely different processing pathways of albumin and other proteins as discussed in detail above. The high capacity sites retrieves filtered proteins and returns them to the blood supply whereas the low capacity site is involved in lysosomal degradation of filtered protein. The capacity of these pathways, particularly the high capacity receptor, can accommodate relatively high filtered amounts of proteins such as albumin due to its high sieving coefficient (Table 1). This basically ensures that the PTC acts as an absolute gatekeeper in determining the level of proteinuria/albuminuria.

**TABLE 1:** A calculation of the amount of albumin filtered by rabbit kidneys (g per day) as compared to potential tubular uptake of filtered albumin by the high capacity receptor. The high glomerular sieving coefficient for albumin of 0.03 is explained in the text. The number of glomeruli in rabbit kidneys comes from Moore and Hellman (1931).

	
$\begin{array}{l} Whole\;kidney\;filtration\;of\;albumin=\\ Glomerular\;sieving\;coefficient\times plasma\;albumin\;concn\times GFR=\\ 0.03\times 30\times 10^{-3}\left(g/ml\right)\times 4.8\times 10^{3}\left(ml/d\right)=4.3\left(g/d\right) \end{array}$	
$\begin{array}{l} Tubular\;uptake=\\ Length\;of\;proximal\;tubule\times number\;of\;glomeruli\times tubular\;uptake\\ \;14\left(mm\right)\times 2.5\times 10^{5}\times 2\times 10^{-9}\times 1.44\times 10^{3}\left(g/mm.d\right)=10.1\left(g/d\right) \end{array}$	

**FIGURE 2:**
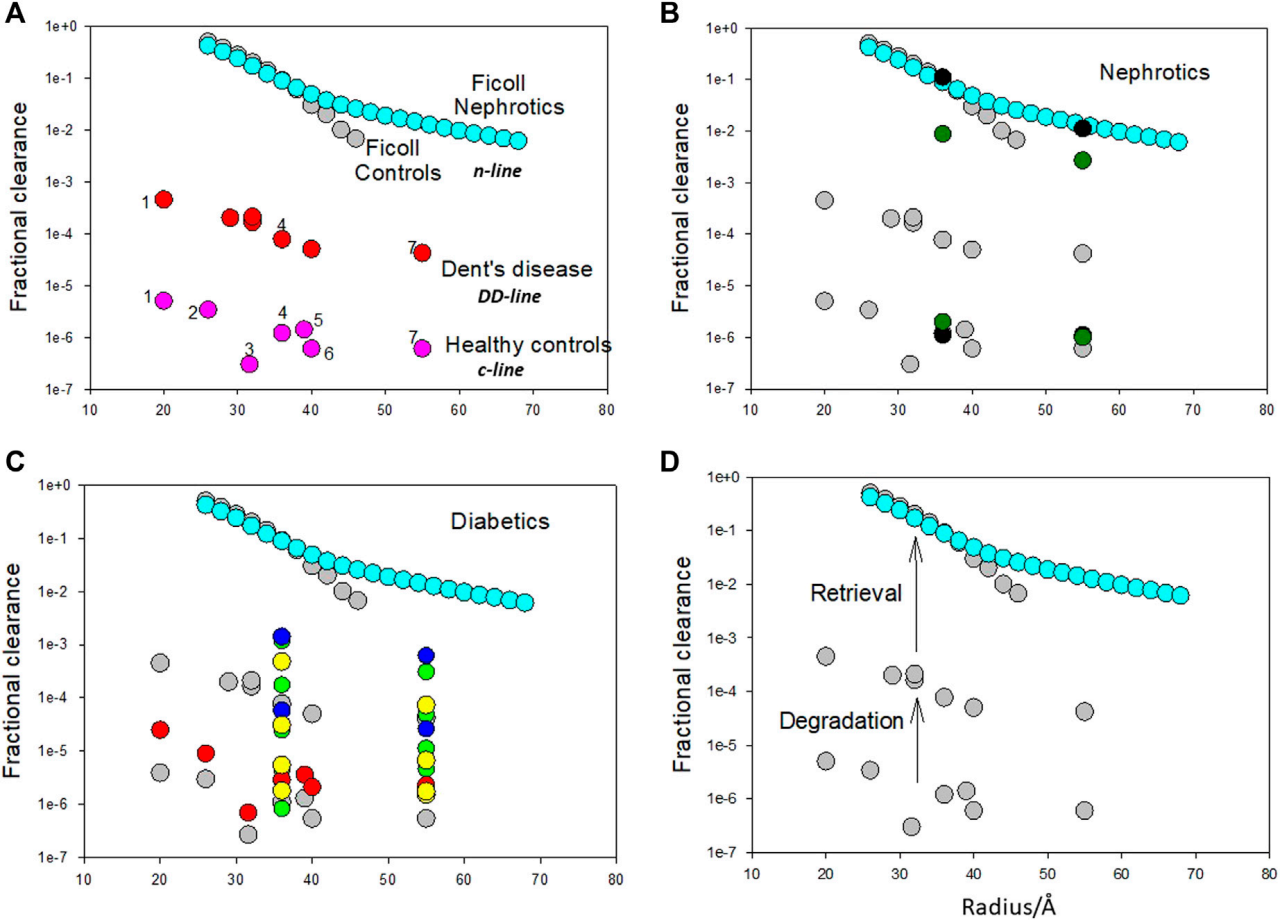
**(A)**. Fractional clearances of various proteins (pI in parenthesis) in nondiabetic Caucasians (healthy controls) where 1 = α1 acid glycoprotein (2.7), 2 = α2 HS glycoprotein (5.4), 3 = α1 antitrypsin (5.0), 4 = albumin (5.0), 5 = hemopexin (5.9), 6 = transferrin (5.5), 7 = immunoglobulin (6.4–9.0) (from Aronoff et al. (Aronoff et al., 1981)) versus molecular radius in Ångstroms (variation is the c-line). Fractional clearances of various plasma proteins with Dent’s disease [from Norden et al. (Norden et al., 2001)] are also presented (dd-line). Fractional clearances of Ficolls as a function of size for nephrotics is from Blouch et al. (Blouch et al., 1997) (n-line). The Ficoll data for radii >45 Å for healthy controls was not included as it appears to be influenced by cellular uptake post-filtration [Vuchkova et al. (Vuchkova et al., 2014)] which does not occur in nephrotic states. It is apparent that there is a remarkable parallelism in the data as a function of radius suggesting that the major differences in the range of fractional clearances are the result of common processing of these proteins by the kidney. **(B)**. Fractional clearances of albumin and IgG in healthy (lower set of fractional clearances) and nephrotic humans (higher set of fractional clearances) (filled black circles- Blouch et al. (Blouch et al., 1997); filled green circles-Guasch et al. [Guasch et al., 1993)] overlaid on important comparative data (in turquoise and grey) from Figure 1A. **(C)**. Fractional clearances of proteins in diabetic patients. Filled red circles represent data from diabetic Pima Indians [Aronoff et al. (Aronoff et al., 1981)]; filled blue circles represent two groups of insulin-dependent diabetes mellitus patients with increasing albumin excretion rate (Scandling and Myers, 1992); filled yellow circles represent five groups of Type I diabetic patients with increasing albumin excretion rate (Deckert et al., 1993); filled green circles represent four groups of Type I diabetic patients with increasing albumin excretion rate (Deckert et al., 1988). **(D)**. Proposed mechanism for bi-phasic processing of filtered protein processing. The degradation pathway utilizing the megalin/cubilin receptor complex is a relatively high affinity pathway that degrades a small amount of the filtered protein. This pathway may function to remove denatured proteins or proteins not taken up by the retrieval pathway. Loss of protein by the degradation pathway does not result in serious clinical features such as hypoproteinemia. The retrieval pathway accounts for the uptake of the majority of filtered proteins and returns them to the blood supply intact as demonstrated for albumin. Loss of the retrieval pathway, as in nephrotic states, results in hypoproteinemia; the albumin to IgG fractional clearance ratio remains essentially unaltered. Adapted from Comper et al. (Comper et al., 2016).

These binding sites can also be correlated with fractional clearances of proteins in healthy and diseased states (Figure 2). The fractional clearance of a wide variety of different proteins, including albumin, in healthy human controls is shown in Figure 2A; their size dependence is relatively weak extending just about an order of magnitude over a radii range of 20–55 Å (c-line). Inhibition of the degradation pathway can occur with the loss of ClC-5 chloride channel in the proximal tubule in Dent’s disease (DD) which impairs membrane traffic of megalin and cubilin receptors (Christensen et al., 2003). Norden et al. (Norden et al., 2001) examined the fractional clearances of different proteins in DD. They obtained very similar results to Aronoff et al. (Aronoff et al., 1981) for healthy controls except the fractional clearances were 2 orders of magnitude higher; they exhibited the same variation with size having a weak size dependency. For albumin, the transition of low fractional clearances in the range of 10^−5^–10^−6^ to 10^−4^ can be quantitatively related to the J_ALB_^max^ and K_m_ of the low capacity pathway (Comper et al., 2008) and its inhibition. The degradation pathway is a minor cause of proteinuria and its inhibition does not result in hypoproteinemia.

In nephrotic states both albumin and IgG clearances approach those obtained with Ficolls (Figure 2B) (n-line). The other striking feature about the n-line is the universal agreement and invariant results in the Nephrology literature that the fractional clearance of inert transport probes like Ficolls, of similar radius to albumin, are not affected in nephrotic states as shown in Figure 2A (Vuchkova et al., 2014). This means that the increment from the dd-line to n-line is not a size selective defect. This is confirmed by the ratio of fractional clearances of albumin to IgG; there is essentially no change (Figure 2B).

Figure 2C includes a range of protein fractional clearance data for diabetic patients. Most of the data lies in the range where the degradation pathway would operate (below the DD line for protein clearance) indicating that proteinuria/albuminuria in diabetic states is predominantly a metabolic event associated with the integrity of the protein being excreted (Osicka et al., 1997; Osicka et al., 2000; Russo et al., 2002; Comper et al., 2008; Vuchkova and Comper, 2015).

A dilemma then arises to explain albuminuria and loss of plasma albumin in nephrotic states; the endocytotic megalin/cubilin system does not explain it (Weyer et al., 2018) but neither does a change in the permeability of the glomerular filtration barrier (GFB) nor the ‘loss of GFB charge’ (see below). The transition from the dd-line to the n-line has been interpreted in our laboratory as being governed by the retrieval pathway (Figure 1 and Figure 2D) where filtered proteins are taken up by the proximal tubule and returned to the blood supply intact. For albumin, the transition of low fractional clearances in the range of 10^−5^–10^−6^ to 10^−2^–10^−1^ in nephrotic states (Figure 2B) can be quantitatively related to the J_ALB_^max^ and K_m_ of the high capacity retrieval pathway (Comper et al., 2008) and its inhibition. Inhibition of the retrieval pathway is a major cause of nephrotic proteinuria and results in hypoproteinemia.

Figure 2D features the proposed by bi-phasic nature of albuminuria/proteinuria associated with the degradation pathway and retrieval pathway. Filtered proteins are retrieved by the retrieval pathway and returned to the blood supply. Small quantities (<5%) not retrieved undergo degradation by the degradation pathway prior to excretion of the degradation products (Figure 1). Inhibition of the degradation pathway can be quite independent of the retrieval pathway (Russo et al., 2002; Comper et al., 2008). The biphasic processing is entirely consistent with the two albumin binding sites described by Park and Maack (Park and Maack, 1984; Russo et al., 2002; Comper et al., 2008) (Figure 1).

### Variable proteinuria/albuminuria

Variation in albuminuria, without hypoalbuminemia, is common in diabetic kidney disease and in other diseased states. Figure 2C demonstrates that most of the data lies in the range where the degradation pathway would operate (below the DD line for protein clearance) indicating that albuminuria/proteinuria in diabetic states is predominantly a metabolic event associated with the integrity of the albumin being excreted (Osicka et al., 1997; Osicka et al., 2000). This is often confused for a change in GFB permeability for albumin and IgG but in actual fact it is a change in the balance of intact material versus degraded material being excreted; urine assays used for the detection of protein generally only measure the intact material, not the degraded material.

In contrast, nephrotic albuminuria associated with hypoalbuminemia is accompanied by protein fractional clearances that approach the fractional clearances of Ficoll. Partial nephrosis, as manifested as intermediate fractional clearances between the dd-line and n-line are generally not seen for non-diabetic nephrotic states in humans (Figure 2B). The same is observed for nephrotic states in rats and mice (Russo et al., 2002; Comper et al., 2008; Comper et al., 2016). Therefore, the evolution of nephrotic proteinuria/albuminuria is seen as a ‘saltation effect’ rather than the gradual change seen in proteinuria/albuminuria in normo-proteinemic states.

The interesting question arises as to whether there is inhibition of the degradation pathway in nephrotic states. In relative terms there is; there is a marked predominance of intact material excreted over that of degraded material (Greive et al., 2001). Yet, if you consider the degradation in terms of the capacities of the Park and Maack binding sites then there is no inhibition within the error analysis. Greive et al. (Greive et al., 2001) found essentially the same excretion of degradation product from filtered albumin, or transferrin or IgG as measured in healthy rats as compared to nephrotic rats with either anti-GBM disease or puromycin aminonucleoside (PAN) nephrosis.

### Is nephrotic proteinuria the same in acquired kidney disease as it is in genetic perturbation of the GFB?

Not many studies have characterized this. Russo et al. have demonstrated no change in size selectivity, and inhibition of the retrieval pathway occurs in CD2AP KO mice (Russo et al., 2013). Relative inhibition of the degradation pathway is apparent but the excretion rate of degradation products again is not too dissimilar to that observed in healthy mice. These characterizations overlap significantly with those in nephrotic acquired kidney disease.

## Biophysical factors affecting glomerular filtration

It is apparent in Figure 2A that no protein appears as an outlier in healthy controls in spite of at least a 3-fold variation in pI, radius and molecular weight and a 100-fold difference in plasma concentrations. What is remarkable about these protein fractional clearance data is that they are 4-5 orders of magnitude lower than the fractional clearance of their biophysically inert counterparts, Ficolls of the same radii. We discuss here that there is no biophysical explanation that comes close to explaining these differences. While several glomerular centric biomechanical guesses have been put forward such as filtration build up or concentration polarization (Robinson and Walton, 1989; Fissell, 2020) or repetitive compression (Benzing and Salant, 2021) there has been no attempt to quantitate these in relation to the fractional clearance differences between healthy controls and nephrotic patients.

### What role does charge selectivity play in ultrafiltration?

The enthusiasm for generating a charge selectivity concept was derived from the original micropuncture studies (see below) that suggested that albumin was essentially impermeable to the GFB.

#### Binary interaction of albumin with glycosaminoglycans

The best test for charge selectivity is through direct analysis of albumin interaction with glycosaminoglycans (GAGs). This interaction can be of two types. An equilibrium interaction is one where excluded volume is relevant; this will be important in estimating the ability of albumin to enter the GAG-containing charged regions of the GFB. The second interaction is a relative mobility interaction; this will be important in determining transport within the GAG-containing charged regions of the GFB.

#### Quantitation of the equilibrium interaction

In the 1930s and 1940s there was the realization by a number of polymer physicists like Flory, Huggins and Staudinger (see Kurata (Kurata, 1982)) that equilibrium interactions between polymer solutes can be measured by osmotic pressure analysis. They exhibit ‘non-ideal’ osmotic behaviour from van’t Hoff’s law that can be described as

$$\Pi_{a}^{*}/RT=C_{a}/M_{na}+A_{2a}^{OS}C_{a}^{2}+A_{3a}^{OS}C_{a}^{3}+......$$

Here *C*_*a*_ is the mass concentration of the solute *a*, *M*_*na*_ is the number average molar mass of the solute *a* and *A*_*2a*_^*OS*^ and *A*_*3a*_^*OS*^ are the osmotic second and third virial coefficients. The second virial coefficient is an average of all the binary interactions for all solute molecules and will generally dominate quantitatively for polymer concentrations up to 50 mg ml^−1^. For a mix of polymer solutes *a* and *b* the osmotic pressure can be described as

$$\Pi_{ab}^{*}/RT=C_{a}/M_{na}+C_{b}/M_{nb}+A_{2a}^{OS}C_{a}^{2}+A_{2b}^{OS}C_{b}^{2}+A_{ab}^{*}C_{a}C_{b}+......$$

where A*_ab_ is the interaction coefficient between *a* and *b.* The second virial coefficient can be described in modelling terms as steric exclusion of a compound from part of a solution by the presence of another identical molecule that leads to an increase in its concentration in the remaining space. Similarly, the interaction coefficient will describe the excluded volume between different molecules, in this case polymers *a* and *b*. The magnitude of the exclusion is intimately related to the size and shape of the interacting molecules.

Therefore, osmotic pressure measurements are an excellent way of understanding the equilibrium interactions of anionic GAG chains with themselves and with other polymers such as albumin. The second virial coefficient of GAGs has been measured extensively in the 1960s and 70s by Cleland, Laurent, Matthews, Ogston and Preston (Comper and Laurent, 1978). The interaction coefficient of GAGs or uncharged dextran with albumin under physiological ionic conditions have also been measured by the same authors (Ogston and Preston, 1966; Ogston and Silpananta, 1970; Shaw and Schy, 1977).

Table 2 presents potential influence of these interactions on the GSC of albumin across the GFB. The magnitude of the problem to be addressed is expressed in the initial rows. Whether one examines the comparison of transglomerular transport of dextran as compared to albumin or examines the transglomerular transport of albumin in nephrotic conditions the effect is huge in biophysical terms; bordering on a percent change of 5000. What this is saying is that beyond steric exclusion of albumin by the GFB there is a force, claimed in the Nephrology literature to be electrostatic, that is giving rise to this enormous resistance to albumin entry and transport in the GFB. However, the other data in the Table suggests that this cannot possibly be due to interactions of albumin with the GAG chains of the GFB. Specifically, it is apparent that the second virial coefficient of GAG chains are not very sensitive to ambient ionic strength indicating that charge effects generated by them in self interactions are relatively small. The actual value is also relatively independent of the type of GAG chain (Comper and Laurent, 1978). The relatively small differences are also seen with the comparison of albumin interacting with an uncharged polysaccharide versus a GAG.

**TABLE 2:** Simplified representative data to calculate the percent change of different parameters possibly affecting the glomerular sieving coefficient (GSC) of albumin.

	Magnitude	Percent change
GSC dextran equivalent radius to albumin (Comper and Russo, 2009)	0.03	4900
Apparent GSC albumin healthy rats (Tojo and Endou, 1992) (micropuncture)	0.0006	
GSC albumin nephrotic rats (Comper and Russo, 2009)	0.03	4900
Apparent GSC albumin healthy rats (Tojo and Endou, 1992)	0.0006	
A_2_^os^ GAG physiological ionic strength/units (Comper and Laurent, 1978)	1.3–3.3	27
A_2_^os^ GAG high ionic strength/units (Comper and Laurent, 1978)	1.3–2.2	
A* normalized^§^ albumin interaction with uncharged polysaccharide, physiological ionic strength (Comper and Laurent, 1978)	1.0	0
A* normalized^§^ albumin interaction with GAG (Ogston and Preston, 1966; Ogston and Silpananta, 1970; Shaw and Schy, 1977)	1.0	

Normalization was performed by using the ratio of the interaction coefficient to the second virial coefficient of the polysaccharide.

#### Quantitation of transport interaction

The influence of GAGs on the transport of albumin is unremarkable considering the magnitude of the effects in the kidney. At a GAG concentration of 20 mg ml^−1^, albumin transport may be reduced by 50% (Ogston et al., 1973).

#### Model systems

In biophysical terms, the apparent GFB effect on albumin transport is an extraordinary phenomenon. Efforts to construct *in vitro* systems to mimic the entry problem as suggested by the data in Table 2 have failed (Deen and Smith, 1982; Smit and Comper, 1996). Other types of experiments thought to be due to charge (phenomenology) have now been worked out to be due to other confounding effects (Comper and Russo, 2009).

### Physiological studies utilizing inert charged transport probes-exploring charge selectivity

When confounding factors such as cell uptake, metabolic degradation, binding to plasma proteins and other components are eliminated it becomes clear that charged inert transport probes of equal or greater negatively charged valence to albumin are not affected by charge selectivity. This has been demonstrated for dextran sulfates (Comper et al., 1994; Vyas et al., 1996), carboxymethyl Ficolls (Greive et al., 2001; Guimarães et al., 2003) and negatively charged dextran and hydroxy ethyl starch (Schaeffer et al., 2002). These experiments demonstrate unequivocally that glomerular charge selectivity or glomerular electrochemical phenonema do not exist.

### Are biochemical alterations in the fixed charge content of the GFB important in governing albuminuria/proteinuria?

#### Glomerular basement membrane

The identification of heparan sulfate in the glomerular basement membrane (GBM) (Kanwar and Farquhar, 1979) provided evidence to support the concept that the GFB acts as a charge selective barrier to negatively charged molecules like albumin. Those early reports led many investigators to focus their investigations to study whether changes in the amount of heparan sulfate and other GBM GAGs were correlative to the level of albuminuria/proteinuria present in disease. Unfortunately, the correlations were weakened by the lack of quantitation of how much heparan sulfate or GAG was present in the GBM. Subsequent genetic perturbations to affect heparan sulfate (Chen et al., 2008; Sugar et al., 2014; Khalil et al., 2022) demonstrated that the animals did not develop proteinuria. There still remains the question that it may not be the level or composition of the GAGs in the GFB but their micro-organization through their involvement in hypothetical charged pores or channels. These questions have been addressed in the previous section which would demonstrate that these specialized pores do not exist.

#### Endothelial glycocalyx

The endothelial glycocalyx has also been targeted as a factor having significant influence in ultrafiltration (Ballermann et al., 2021). This region is >95% water yet there are claims by various groups (Ballermann et al., 2021; van den Berg et al., 2019; Butler et al., 2020) that the endothelial glycocalyx plays a critical role in albumin filtration across the GFB. The biophysical studies referred to above, if interpreted in light of the known of the equilibrium and dynamic interactions of albumin with GAGS, would suggest that its size exclusion function would only be capable of limiting the passage of molecules far greater in size than that of albumin (Comper, 2022). Evidence supporting this concept can also be extrapolated by *in vivo* labeling studies of the glomerular basement membrane for laminin (Abrahamson, 1986a; Abrahamson, 1986b; Couchman et al., 1993; McCarthy et al., 1993) and heparan sulfate proteoglycan (Stow et al., 1985; Miettinen et al., 1986). The primary focus of the aforementioned studies was not to determine the porosity/size exclusion capability of the GFB but to use tagged antibodies to immunolabel the GBM. The fact that peroxidase-labeled antibodies (MW IgG = 150 kDa; MW horseradish peroxidase = 40 kDa) were shown to readily cross the GFB endothelial layer to label the entire glomerular basement membrane or both lamina rarae would strongly suggest that the molecular weight cutoff with regard to the size exclusivity of the endothelial cell layer and the GBM itself to be far greater than what has been postulated by many investigators over the years.

### Alternative explanations for well known studies purporting charge-mediated interactions in the kidney

#### Deen-Brenner model using dextran sulfate

**FIGURE 3:**
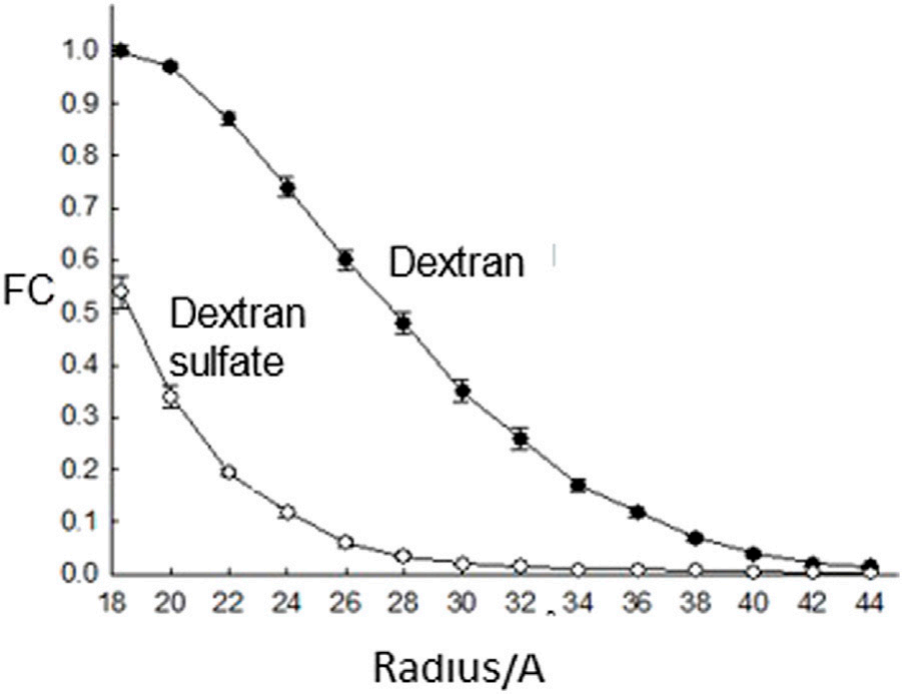
The fractional clearance (FC) of dextran sulfate and neutral dextran as a function of molecular radius as studied in rats (Chang et al., 1975a).

Perhaps the major piece of evidence, presented in all the medical text books, for glomerular charge selectivity is the decreased fractional clearance of dextran sulfate versus dextran (apparent charge selectivity) with the same molecular radius (Figure 3). Micropuncture studies had established that there was essentially no tubular reabsorption for these filtered probes (Chang et al., 1975a) and that it was assumed these probes were inert. However, the latter assumption was proven incorrect with the finding that dextran sulfate is metabolically desulfated during transglomerular transport (Comper et al., 1994). Apparent charge selectivity could be destroyed simply by increasing the level of circulating dextran sulfate or lowering the degree of sulfate substitution from 1.7 sulfates per glucose residue to 1.0 sulfates per glucose residue (Vyas et al., 1996). As the concentration of circulating dextran sulfate was increased or its degree of sulfate substitution decreased, the level of desulfation also decreased. A model to explain these results was generated through dextran sulfate uptake to glomerular endothelial cells (Vyas et al., 1996).

#### Haraldsson model using the cold IPK

Apparent charge selectivity was measured in the cold isolated kidney perfusion system (cIPK) through the difference in the fractional clearance of albumin and Ficoll with the same hydrodynamic radius of 36 Å (Ohlson et al., 2001; Jeansson et al., 2006; Jeansson and Haraldsson, 2006; Jeansson et al., 2009). In those studies, fractional clearance differences are near maximal at very low glomerular filtration rates (GFR) (<10% of normal). However, when the operating GFR was increased to 50% normal the fractional clearance differences were shown to decrease by 90%. Extrapolation to normal GFR would indicate that there are no differences at all (Comper and Russo, 2009). The conclusion one can draw from these studies is that the apparent charge selectivity is massively dependent on GFR whereas genuine charge selectivity should be completely independent of it. Clearly there are other non-charge related factors determining this GFR dependence in fractional clearance (Comper and Russo, 2009).

#### Moeller electrokinetic model

It was already established that there are no significant charge effects on transglomerular transport across the GFB. It means that any other theory is redundant. This did not stop Moeller et al. (Hausmann et al., 2010) who proposed a model that streaming potentials generated by transient charge separation induced by fluid flow on the fixed negative charges of the GFB could conceptually provide resistance to albumin transglomerular transport. It should be noted that since that work was published 12 years ago there has been no follow-up whether this electric potential is a major force in albumin transport. There were attempts at theoretical substantiation but this is critically determined by the intrinsic electrophoretic mobility of albumin which was calculated to be 4.7 × 10^−8^ m^2^ s^−1^volt^−1^ (Hausmann et al., 2010); but this is unrealistic given that the measured value of albumin electrophoretic mobility in free solution (Westphal and Priest, 1955) is much lower at 5.8 × 10^−9^ m^2^ s^−1^volt^−1^ and will be definitely lower within the GFB. Overall, this makes the streaming potential effect a minor player influencing albumin transglomerular transport.

### Size selectivity

Early studies by Brenner’s group (Chang et al., 1975b; Bohrer et al., 1979) established that for dextrans and Ficolls, used in size selectivity fractional clearance studies (radius range ∼20–40 Å), there was essentially no tubular reabsorption of filtered material as measured by micropuncture studies; that changes in fractional clearance could be directly related to changes in transglomerular transport. Interestingly, there has been no published work that has seen any change in transport of these probes in proteinuric/albuminuric states^1^.

#### Large pores in nephrotic states?

The same could not be said for inert probes with radii >40 Å. They are most often to be seen to increase in clearance in proteinuric states which led to the concept of large pores being generated. The confounding problem is whether in healthy controls there is tubular uptake of these relatively large probes (which were not tested by micropuncture) which may be inhibited in nephrotic states.

In order to address this we examined (Vuchkova et al., 2014) plasma elimination rates of uncharged polydisperse Ficoll (radii range: 35–85 Å) and two globular ^14^C-labeled proteins, albumin (radius: 36 Å) and IgG (radius: 55 Å), in control and in PAN nephrotic Sprague-Dawley rats. The plasma elimination rates were then matched to the urinary excretion of these labeled materials. In control rats albumin and IgG plasma retention rates were identical and far enhanced compared with the retention rates of inert transport markers of equivalent hydrodynamic radius; their elimination rate corresponded to the elimination of a 75 Å radius Ficoll. In PAN, they were eliminated as ∼36 and ∼55 Å radii Ficoll, respectively, equivalent to their hydrodynamic radii. In contrast, there was no comparable increase in the elimination rate of Ficoll in PAN. The total plasma clearance of Ficoll in control and PAN rats and the urinary clearance in PAN rats were essentially the same for all radii. On the other hand, the urinary clearance of >45 Å radii Ficoll in controls was considerably lower with increasing radii, demonstrating a postfiltration cellular uptake in controls, which, when inhibited in nephrotic states, would account for apparent large pore formation.

Relevant to understanding these clearances is the notable observation of Rippe et al. (Öqvist et al., 1999) that in nephrotic syndrome caused by PAN in rats, it was only the kidney vasculature that was affected and not extrarenal microvascular beds. This means that the remarkable increase in fractional clearance of proteins is specifically related to changes in the kidney. This would be a feature consistent with the inhibition of the retrieval pathway and the unique features of the nephron system.

The fact that in the nephrotic condition albumin is cleared as a 36 Å molecule is supported by the finding that the loss of plasma albumin to give rise to hypoalbuminemia is quantitatively accounted for by the appearance of albumin in the urine (Greive et al., 2001; Koltun and Comper, 2004; Koltun et al., 2005; Vuchkova et al., 2014; Comper et al., 2016).

The failure of the old Nephrology concept of GFB impermeability to albumin in healthy controls and the ‘development of filtration of nephrotic levels of albumin in nephrotic states’ is no more exemplified than in a recent publication by Christensen’s group (Ren et al., 2020). Cubilin and megalin are claimed to be involved in Park and Maack’s high affinity albumin binding site (degradation pathway) and now megalin for the low affinity high capacity site (retrieval pathway). They speculate the low affinity high capacity site is for albumin recovery in nephrotic states. This doesn’t happen. As we discussed above, mass-balance analysis of albumin in nephrotic hypoalbuminemic states demonstrated that albumin in not taken up by the tubules and behaves like Ficoll (Koltun et al., 2005). Therefore, the recovery/retrieval process is more relevant for the non-diseased state as there is normally leakage of nephrotic levels of albumin.

## Measurement of the glomerular sieving coefficient-troubleshooting the old studies

### Micropuncture-difficulties due to time dependence and retrieval pathway

Perhaps the single most influential experiment that has formed the basis of thinking about glomerular ultrafiltration is from the original studies on micropuncture (Wearn and Richards, 1924), performed almost 100 hundred years ago. Wearnand Richards demonstrated that the filtrate was essentially protein free, not just albumin free. There started the dogma in Nephrology that the glomerular filter was essentially impermeable to albumin and other plasma proteins. It also became apparent that the expected low concentrations of albumin in the primary filtrate influenced data selection in later micropuncture and 2-photon studies (see below).

Confidence in the micropuncture technology was enhanced by Brenner’s group (Chang et al., 1975a; Chang et al., 1975b; Bohrer et al., 1979) who demonstrated that the GSC of inert probes like dextran and Ficoll were the same as their fractional clearance demonstrating that their reabsorption by the PTC was negligible. Then something strange happened. Efforts at measuring the GSC for albumin, through micropuncture, were met with difficulty as highly variable results were obtained. The expectation was that the GSC would be low, so when high values were obtained they were discarded on the basis that it must be some kind of contamination from extra-tubular sources (Oken and Flamebaum, 1971) (note that no such contamination or data elimination was ever described for studies on dextran and Ficoll). In spite of all the micropuncture studies performed by Brenner’s group they never reported the results of albumin GSC by micropuncture. Furthermore, Oken and Flamebaum did not find contamination of albumin GSC in nephrotic states (Oken and Flamebaum, 1971).

The micropuncture studies of Tojo and Endou (Tojo and Endou, 1992) exemplify the problem. Basically flow was stopped in a nephron and fluid fractions of proximal tubule filtrate fluid near the GFB were serially collected to yield 4 fractions. The first fraction collected would have given a GSC for albumin of 0.06 (not dissimilar from Ficoll fractional clearance in controls and nephrotics and also albumin fractional clearance in nephrotics, Figure 2). They thought this high value was due to contamination. Tojo and Endou took another 3 fractions of the fluid over a period of time and eventually came to a constant albumin concentration yielding a GSC of 0.0006 which they felt was correct. So the questions arise; where did the initial contaminating albumin ultimately go as it was not seen in the final fraction collected? If it was taken up by proximal tubule cells, does it mean that if they had waited they may not have seen the contaminating albumin? Or were these results simply normal leakage of albumin due to lack of GFB charge and retrieval of albumin over time?

Our interpretation of these data is that the earliest sample taken by Tojo and Endou probably reflected the true GSC of albumin and that over time albumin was being continually removed from the stationary filtrate by both the retrieval pathway and the degradation pathway in the PTC.

### Glomerular uptake studies

Rippe and coworkers (Lund et al., 2003; Rippe et al., 2006; Rippe et al., 2007) used a glomerular uptake technique to determine the GSC of albumin of 0.0006. They used an 8 min infusion into plasma of ^125^I-labelled protein. After infusion, a whole body vascular washout was started with heparinized horse serum via the jugular vein at a rate of 20 ml min^−1^ for approximately 8 min. The trichloroacetic acid (TCA) precipitable urine and cortical tissue (not the inner medulla) were included in radioactivity measurements. The overall problems with the glomerular uptake technique is that it does not include the non-TCA-precipitable radioactivity in the urine derived from renal degradation of filtered material, does not include the material that is bound to the surface of the PTCs that could be exchanged with unlabelled albumin during the washout, fails to prove the assumption that there was no washout of retrieved albumin in the PTCs during the 8 min washout period and is unable to account for retrieved albumin that had already been returned to the blood supply during the 8 min infusion.

### 2-Photon microscopy-known GSC transport calibrants rarely used

Once it was realized that charge selectivity was not a factor in governing the transglomerular transport of albumin there was a compelling need to measure the albumin GSC directly. We expected the GSC to be high yet when the first two photon measurements of the albumin GSC of 0.03 were published (Russo et al., 2007) there was an outcry in terms of their veracity. It became clear that many issues can affect the measured the two photon GSC; these have been investigated extensively and thoroughly by Sandoval, Molitoris and others (Sandoval et al., 2012; Sandoval et al., 2014). Depending on the settings of the microscope, investigators can get any value of GSC. As it turns out some two photon investigators have chosen to publish the very low values of albumin GSC (Peti-Peterdi, 2009; Schießl et al., 2015; Butler et al., 2019) (no doubt influenced by the micropuncture/charge selectivity dogma in the kidney field) without any qualifying data, particularly the use of transport calibrants of known GSC. The criticism that can then be levelled at all these studies is ‘how do they know they have measured the correct GSC?’ It is absolutely necessary for two photon studies that adequate transport calibrants of known GSC be used to ensure the correct settings on the microscope. The only studies that have done this is by Russo et al. (Russo et al., 2009) and Sandoval et al. (Sandoval et al., 2012) and accordingly they have obtained the relatively high GSCs for albumin. These GSC values have been confirmed by a body of work over the years (Wagner et al., 2016; Sandoval et al., 2019; Molitoris et al., 2022).

These studies do provide what was originally expected when cell-mediated effects are not involved and knowing that charge selectivity is not a factor; the GSC of 36 Å radius Ficoll in healthy control humans = GSC of 36 Å radius Ficoll in nephrotic humans = the GSC of albumin in nephrotic humans = GSC of albumin in healthy controls (rats).

### Overload albuminuria-general agreement among all published work

There is an interesting set of studies that induces reversible nephrotic-like albuminuria; that is overload proteinuria as studied in rats (Davies et al., 1978; Bliss and Brewer, 1985; Weening et al., 1987; Lemley, 1993; Koltun and Comper, 2004; Wagner et al., 2016). In overload proteinuria, an artificial small increase in plasma protein (∼20%) induced by iv injection of albumin solution in healthy rats can generate profound and disproportionate increases in the urinary excretion of protein (maximal 7-fold increase in total protein excretion, 200-fold increase in intact albumin excretion (see also (Wagner et al., 2016)) and an increase in the excretion of other plasma proteins such as transferrin (Koltun and Comper, 2004)) without any significant change in size selectivity (Koltun and Comper, 2004) or GSC for albumin (Wagner et al., 2016). ‘Nephrotic-like’ albuminuria and proteinuria can occur by simply increasing the plasma albumin concentration by a very small amount. The rat removes this excess albumin/protein in less than 3 days. These results have been interpreted in the following way; these levels of excretion would never occur megalin/cubilin mediated degradation pathway but rather through a saturable receptor (retrieval pathway) that is unable to fully accommodate the extra filtered albumin due to its handling of endogenous filtered albumin; the albumin not taken up by the receptor is excreted into the urine. These types of studies reveal the finely tuned balance between albumin filtration rate, proximal tubule uptake and the concentration of filtered material; in healthy controls it is basically operating at maximal capacity and any further perturbation tips the system into an overflow situation where the albumin is readily excreted. In the Koltun and Comper (Koltun and Comper, 2004) study, an average increment in plasma protein content of ∼10 mg ml^−1^ (day 3), due to albumin overload, yielded an increment of ∼360 mg protein day^−1^ in excretion rate which corresponds to a protein fractional clearance of ∼0.02. In a system where glomerular permeability is essentially unaltered and with endocytotic mechanism by PTCs being nearly saturated but with normal fragmentation still occurring, the results imply that the GSC is at least an average of 0.02 for all glomerular-filtered protein, the majority of which is likely to be albumin. Again, this is in agreement with the two photon studies described above. It is notable that overload proteinuria would never occur if the only tubular uptake was the low capacity degradation pathway associated with a very low GSC.

The immediate albumin filtrate concentration in the rat will be ∼1 mg ml^−1^ yet the results in Figure 1 would suggest that at this concentration the high capacity receptor (albeit for rabbit tubules) is nowhere near saturation. The reason is that the real protein concentration *in vivo* is going to be considerably higher due to the presence of non-albumin plasma proteins that are also filtered and retrieved. If we assume that albumin is approximately 50% of the total protein present then we should be looking at P_ALB_ near 2 mg ml^−1^ (assuming albumin is just like other plasma proteins here) where the receptor is far closer to saturation. The transition from normal filtration *in vivo* to overload/saturability is very tight and much tighter than exemplified by the line of best fit of the upper inflexion of the uptake curve shown in Figure 1.

### Tertiary structure of albumin

The functional interplay of the retrieval and degradation pathways has still to be worked out. We have noted, however, that the tertiary structure of albumin, beyond simple size and charge, is a critical determinant for albumin processing by the retrieval pathway by the kidney (Clavant and Comper, 2003). It suggests that a specific albumin-recognition event by the kidneys is critical to normal renal handling of albumin otherwise the molecule is not retrieved but is disposed of by the degradation pathway.

### Summary of GSC values obtained from different technologies where the confounding retrieval pathway has been minimized

Table 3 summarizes the albumin GSC data as determined in rats that we judge as valid. All the techniques give similar values in the range of 0.02–0.06. These are the values expected when size selectivity alone is the primary biophysical factor governing transglomerular transport and where charge selectivity is non-existent. The relatively high GSC values are entirely consistent with the original observations by Ryan and Karnovsky (Ryan and Karnovsky, 1976) that albumin can transverse the GFB relatively quickly.

**TABLE 3:** Summary of albumin GSC values obtained in healthy rats by different methods where the confounding retrieval pathway has been minimized.

Method	GSC	Reference
Early sample taken in micropuncture	0.06	Tojo and Endou, 1992
2-photon microscopy	0.03	Russo et al, 2007
Overload proteinuria	0.02	Koltun and Comper, 2004
Pulse chase of labelled albumin into renal artery	0.04	Eppel et al, 1999
Denatured albumin	0.05	Clavant and Comper, 2003

## Cellular involvement in protein filtration, retrieval and excretion

### Retrieval pathway

#### Albumin studies

Albumin pulse-chase studies in the renal artery demonstrated that the time taken to observe the appearance of retrieved albumin in the renal vein was 80–160 s (Eppel et al., 1999). Transcytosis within the PTC of vesicles filled with retrieved filtered albumin can occur within seconds (Russo et al., 2007; Sandoval et al., 2012). In the original studies of Park and Maack (Park and Maack, 1984) the retrieval pathway would not have been observed as the albumin concentration in the perfusate was too low so it would preferentially be taken up by the high affinity degradation pathway. Limited studies by our group (Zhou et al., 2013) have demonstrated that perfusing isolated rabbit tubules with 2 mg ml^−1^ albumin resulted in transcytosis of intact albumin (as determined by chromatography with <3% degraded material) (three different experiments gave values of ng mm tubule^−1^.min^−1^ of 4.4, 20.0 and 2.2 where uptake studies of Park and Maack (Park and Maack, 1984) would predict a value of 2.2); this was not classic fluid phase as demonstrated by the lack of inulin uptake in agreement with Park and Maack. We do note that Ficolls and dextrans (radii >40 Å) are taken up by proximal tubule cells but not retrieved to the circulation (Vuchkova et al., 2014)^2^.

**FIGURE 4:**
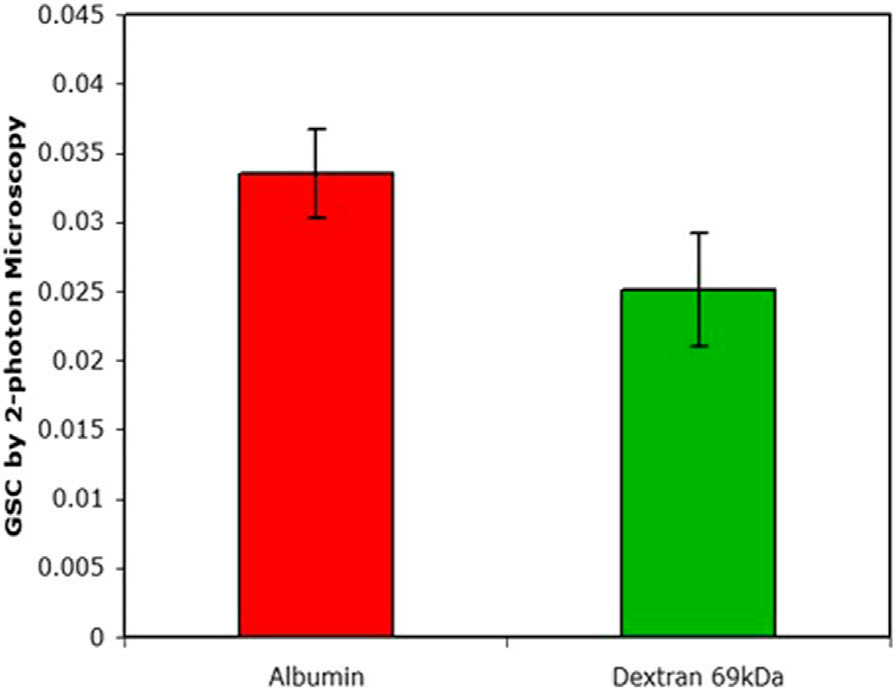
The GSC as determined by 2-photon microscopy. Plasma levels after 24 h of injection were 38% for albumin and <1% for dextran (data from (Russo et al., 2009).

The manifestation of the retrieval pathway is clearly seen in Figure 4. Mono-disperse dextran and albumin have similar GSCs yet their residence time in the circulation is markedly different; after 24 h there was ∼40% of the injected albumin still retained in the circulation whereas there was essentially none of the dextran.

#### Non-albumin plasma protein studies

The remarkable similarity that all plasma proteins exhibit in their fractional clearances, as shown in Figure 2, suggests that they are all processed in a similar way by the PTC. The extensive studies of both albumin and IgG in humans would certainly suggest that this is the case for these two proteins (also albumin, IgG and transferrin in rats).

Protein fractional clearances in analbuminemic rats in healthy controls and nephrotic states demonstrated that non-albumin plasma proteins are processed in the same manner as normo-albuminemic rats (Osicka et al., 2004) suggesting again that the retrieval and degradation pathway are operating on all filtered proteins with molecular weights >30 kDa.

### Degradation pathway-directionality

When Park and Maack (Park and Maack, 1984) perfused tubules at the low albumin concentration of 0.03 mg ml^−1^ they determined that the majority of the degradation products were observed in the bathing solution. Many investigators subsequently interpreted these studies that degradation products produced *in vivo* would be expected to be trancytosed in the PTC and be destined for the circulation. However, we have never seen this *in vivo* (Osicka et al., 1997; Osicka et al., 2000; Russo et al., 2002; Comper et al., 2008; Vuchkova and Comper, 2015); the degradation products are exclusively recycled back to the tubular lumen and excreted. There is the likelihood that the vectorial fate of the degradation products is dependent on the albumin perfusate concentration; when the perfusate concentration was relatively high at 2.2 mg ml^−1^ we did not see those products on the baso-lateral side of the perfused tubule (Zhou et al., 2013).

## Nephrotic proteinuria

We have noted earlier certain characteristics of nephrotic states including 1) the increase in fractional clearances of proteins that approach those of inert probes of the same hydrodynamic radii, 2) no inhibition of the degradation pathway (when considered in terms of the Park and Maack receptors), 3) hypoalbuminemia resulting in a ∼60% loss in plasma protein and 4) associated with only small changes, if any, in the glomerular permeability of inert transport probes (experimental error of the transport measurements is generally no better than within 50%).

### Podocytes potentially initiating nephrotic proteinuria/albuminuria

The results in Figure 2 demonstrate that the fractional clearance of proteins as measured in healthy human subjects increases 10,000–100,000-fold when studied in nephrotic patients. It is remarkable then that genetic perturbation of the GFB can have such a biophysically profound effect (as commonly proposed in the literature) when there is absolutely no clear explanation as to what those biophysical changes are (Kestilä et al., 1998; Tryggvason, 1999; Funk et al., 2020; Benzing and Salant, 2021). The effect of genetic perturbation on glomerular filtration was initially elaborated by Tryggvason et al. (Kestilä et al., 1998; Tryggvason, 1999) when they identified nephrin mutations in the slit diaphragm as being responsible for congenital nephrotic syndrome. There have been many studies since (Tryggvason, 1999; Benzing and Salant, 2021) that have identified various genetic perturbations of the GFB giving rise to altered permeability and even nephrotic proteinuria; how these occur has never been seriously addressed. Tryggvason (Tryggvason, 1999) invoke the ‘importance of size, shape and charge selective properties’ of the GFB in determining the passage of plasma macromolecules whereas Benzing et al. (Benzing and Salant, 2021) invokes additional glomerular centric biomechanical effects on the GFB. As we discussed earlier, none of these speculations quantitatively account for differences in the fractional clearance of plasma proteins from healthy controls to nephrotics as shown in Figure 2. There is no doubt that the etiology of the proteinuria can directly related to the occurrence of the perturbation associated with the podocyte or GFB but there is no evidence that the perturbation itself is directly responsible for altered transglomerular transport properties giving rise to nephrotic proteinuria.

### Do overload proteinuria-like effects have a role in explaining podocyte-mediated nephrotic proteinuria in acquired and genetic kidney disease?- Working models

In speculating the initial events leading to nephrotic proteinuria, we could easily envisage a factor(s) being produced by stressed podocytes^3^ (through genetic perturbation or other means) that inhibit the retrieval pathway downstream. The nature of the factors is not known.

Alternatively we could consider an overload proteinuria effect. The overload effect we described above does not explain nephrotic proteinuria. We could turn the thinking around, however, and say that if there is a very small change in the exclusion properties of the GFB and that the output of albumin filtration across the GFB is increased by 10–20% then this would mimic overload proteinuria (Russo et al., 2013; Comper and Vuchkova, 2021). The changes could conceivably be a result of podocyte stress or deletions in proteins of the GFB altering the transport properties of the extracellular matrix *milieu*. They may be too small to accurately measure directly in terms of GSC or fractional clearance. Yet these small changes would be amplified in terms of protein excretion in a synonymous way as in overload proteinuria. This mechanism sets the basis for understanding how a ‘podocyte related event’ can have a disproportionate influence on albumin/protein excretion determined by the PTCs. There is also an important biomarker that establishes the common phenomenology of overload proteinuria and nephrotic proteinuria; the lack of inhibition of the degradation pathway. This is exactly what occurs in overload proteinuria where there is a saturation of the retrieval receptor but the degradation pathway is still operative (Koltun and Comper, 2004).

It is interesting to speculate how a small increase in GFB permeability can ultimately generate hypoalbuminemia^4^. In both healthy controls and nephrotics the plasma level of albumin will be the net result of albumin synthesis, albumin plasma elimination, kidney retrieval and uptake by proximal tubules. It is assumed that optimal residence in the circulation would occur at the K_m_ value for the retrieval pathway in Figure 1. Any concentration above that corresponding to the K_m_ value may result in some albumin loss into the urine^5^. Therefore, an increase in GFB permeability will have to find a new steady state when the different amount of hypoalbuminemic albumin is presented to the proximal tubule receptors. In this case, further albumin loss will occur and ultimately result in inhibition of the retrieval pathway and maximal hypoalbuminemia.

### Time evolution of nephrosis

We view the initial etiology of the nephrotic state and particularly in nephrotic podocytopathies is a small aberration in the permeability of the GFB. This will result in an overload effect on the PTC with subsequent loss of filtered protein including albumin into the urine. Hypoproteinemia will be set in play. This evolution of nephrosis is probably never measured because, as noted earlier, partial nephrosis is rarely if ever observed. Rather what is seen is the end point of these overload effects where the fractional clearances of proteins particularly albumin and IgG, approach those of their Ficoll counterparts of the same radius and hypoproteinemia has reduced the plasma protein concentration by two-thirds ensuring that overload effects on PTC uptake will not occur (Figure 1). The reason for the release of nephrotic levels of protein into the urine, at this stage, is that the retrieval pathway now appears to be completely inhibited which is consistent with the equivalence of the plasma elimination of proteins with their Ficoll counterparts. The reason for the inhibition is not known although several mechanisms can be envisaged; podocyte factors may be released and operate downstream on the proximal tubular cell to turn off the retrieval pathway or it may be that the retrieval pathway is inhibited by virtue of the hypoproteinemic state. Future delineation of these various mechanisms will determine the primary vascular/glomerular/tubular event that ultimately determines the nephrotic condition.

## Concluding remarks

In terms of understanding proteinuria/albuminuria there has been two schools of thought. The dominant thinking for many years has been that proteinuria/albuminuria is an essentially glomerular problem associated with deficiencies in charge and size selectivity of the filtration barrier; increasing leakage of the GFB will give rise to increasing proteinuria. This idea started when Wearn and Richards (Wearn and Richards, 1924) discovered through micropuncture studies that the ultrafiltrate in normal filtering kidneys contains zero protein. Various studies were then thought to support this impermeability concept. Micropuncture studies were performed in the 1960s but there were problems as the results were time dependent and a significant amount of data eliminated on the basis of untested extra-tubular contamination. In the 1970s the charge selectivity concept evolved particularly to explain why negatively charged albumin did not undergo transglomerular transport due to charge repulsion with the fixed negative charges of the GFB. The concept of ‘large pores’ in the GFB was also introduced to help explain the increased permeability of the GFB in proteinuric states. Finally, over the last 20 years the focus on the integrity of the GFB has been intense with the association of genetic alterations in the components of the GFB and the onset of proteinuria. Glomerular centric mechanisms for the etiology of proteinuria/albuminuria had been proposed but these have been extremely vague and not quantitated; there was certainly no serious effort to understand the role of the PTC in governing the final amount of protein being excreted in urine.

The second school of thought, featured in this review, suggests that many of the conclusions of previous studies are incorrect and the studies themselves misleading. It started when apparent charge selectivity to albumin transglomerular transport was demonstrated not to exist. Dextran sulfate was originally used as a model of negatively charged albumin and its clearance in the kidney could be compared to its uncharged counterpart dextran. It turned out that the results of these clearance studies were confounded by the fact that dextran sulfate is avidly taken up by endothelial cells and desulfated. When the sulfate substitution on the dextran sulfate was lowered to inhibit cell uptake then ‘charge selectivity’ disappeared. Examination of other negatively charged transport probes that were inert and not taken up by kidney cells clearly demonstrated that charge selectivity did not exist. In terms of size selectivity, macro-changes have never been observed and that apparent ‘large pores’ were the result of PTC uptake of probes like dextran and Ficoll with radii >40 Å. If macro-changes in size selectivity do not exist and if charge selectivity does not exist then the GSC for albumin should be similar to uncharged inert probes of similar radius and this is exactly what was discovered in the first direct measurements *in vivo* of the GSC for albumin (Russo et al., 2007). The relatively high GSC meant that albumin leaks slightly across the GFB so it requires retrieval. There now has been a number of studies demonstrating this retrieval by the PTC (Eppel et al., 1999; Tenten et al., 2013; Zhou et al., 2013). In fact, a broader examination would suggest that all plasma proteins in the molecular weight range of 30–150 kDa leak to some extent across the GFB and are subsequently retrieved (Comper et al., 2016). This now points to the importance of the nephron, rather than just the glomerulus, in determining proteinuria. The glomerular filter normally leaks small amount of proteins and its the processing of these proteins downstream by the PTCs that is of paramount importance in governing the amount being excreted and the level of proteinuria. The podocyte and GFB extracellular matrix are sensitive regulators to the amount of protein being filtered; any biochemical/genetic perturbation of the matrix may result in small changes in its environment for transport of filtered proteins that may have a profound effect downstream to saturate/inhibit the uptake of filtered protein in the tubular system. Another feature of the nephron control of proteinuria is that proteinuria/albuminuria itself is not a continuum as it would be expected if it was under purely glomerular control. The biphasic nature of proteinuria/albuminuria was demonstrated by the characterization of the Park and Maack (Park and Maack, 1984) receptors on the PTC; a high capacity receptor associated with retrieval of leaked filtered protein and a low capacity receptor associated with degradation of small amounts of filtered protein.

The scavenger/retrieval role of the PTC now takes on broader dimension. Not only does it retrieve water and NaCl but it also plays a critical role in the retrieval of plasma proteins that are leaked through the glomerular filter.

Finally, when it is reported that albuminuria/proteinuria exists it is probably of one of two forms; it will be either nephrotic with accompanying hypoproteinemia or it will be a reflection of changes in the degradation pathway with more intact material being excreted.

## Footnotes

1 Generally the accuracy of fractional clearance measurements is with 20–50%. Therefore when referring to any macro-changes in clearance, the change would exceed this range. Micro-changes in transport, such as those that may occur in the range <50% are difficult to measure but may play a role in an overload proteinuria effect discussed in the text.

2 The major evidence that the high capacity receptor is not fluid phase uptake is its saturability with a finite Michaelis constant (Figure 1). Saturability can be reflected in overload proteinuria (see below). It is also not related directly to the FcRn as FcRn-deficient or β2M-deficient mice are not proteinuric (Koltun et al., 2005). Inulin used in isolated perfused tubule experiments to detect leakage is not taken up by this receptor (Park and Maack, 1984; Zhou et al., 2013).

3 Stress may arise either through altered biomechanical environment and/or altered extracellular matrix component interaction profile.

4 The issue of when GFB charge and size selectivity are important depends on the context of the discussion. If one is trying to explain the transition of the c-line to the n-line in Figure 2, which has been a major focus in Nephrology for decades, then these concepts collapse as they cannot account for the vast changes in clearances that occur. Paradoxically, in nephrotic states generated by overload effects there is a place for small changes in size and charge selectivity that may contribute to an overall small change in GFB permeability, which in turn, may give rise to nephrotic proteinuria.

5 The data is simply not available to know the efficiency of uptake of albumin/protein by the PTC in relation to what may escape uptake and be excreted in the urine. It may not be necessary to operate at the K_m_ value corresponding to J_ALB_^max^ to attain optimal efficiency in terms of minimizing the material excreted
